# Retrospective Planning Study of Patients with Superior Sulcus Tumours Comparing Pencil Beam Scanning Protons to Volumetric-Modulated Arc Therapy

**DOI:** 10.1016/j.clon.2020.07.016

**Published:** 2021-03

**Authors:** S.-L. Wong, J. Alshaikhi, H. Grimes, R.A. Amos, A. Poynter, V. Rompokos, S. Gulliford, G. Royle, Z. Liao, R.A. Sharma, R. Mendes

**Affiliations:** ∗ University College London Cancer Institute, London, UK; † Department of Clinical Oncology, University College London Hospitals NHS Foundation Trust, London, UK; ‡ Department of Medical Physics and Biomedical Engineering, University College London, London, UK; § Department of Radiotherapy Physics, University College London Hospitals NHS Foundation Trust, London, UK; ¶ Division of Radiation Oncology, The University of Texas MD Anderson Cancer Center, Houston, Texas, USA; || NIHR University College London Hospitals Biomedical Research Centre, London, UK; ∗∗ Saudi Particle Therapy Centre, Riyadh, Saudi Arabia

**Keywords:** Carcinoma, non-small cell lung, proton therapy, X-ray therapy

## Abstract

**Aims:**

Twenty per cent of patients with non-small cell lung cancer present with stage III locally advanced disease. Precision radiotherapy with pencil beam scanning (PBS) protons may improve outcomes. However, stage III is a heterogeneous group and accounting for complex tumour motion is challenging. As yet, it remains unclear as to whom will benefit. In our retrospective planning study, we explored if patients with superior sulcus tumours (SSTs) are a select cohort who might benefit from this treatment.

**Materials and methods:**

Patients with SSTs treated with radical radiotherapy using four-dimensional planning computed tomography between 2010 and 2015 were identified. Tumour motion was assessed and excluded if greater than 5 mm. Photon volumetric-modulated arc therapy (VMAT) and PBS proton single-field optimisation plans, with and without inhomogeneity corrections, were generated retrospectively. Robustness analysis was assessed for VMAT and PBS plans involving: (i) 5 mm geometric uncertainty, with an additional 3.5% range uncertainty for proton plans; (ii) verification plans at maximal inhalation and exhalation. Comparative dosimetric and robustness analyses were carried out.

**Results:**

Ten patients were suitable. The mean clinical target volume D95 was 98.1% ± 0.4 (97.5–98.8) and 98.4% ± 0.2 (98.1–98.9) for PBS and VMAT plans, respectively. All normal tissue tolerances were achieved. The same four PBS and VMAT plans failed robustness assessment. Inhomogeneity corrections minimally impacted proton plan robustness and made it worse in one case. The most important factor affecting target coverage and robustness was the clinical target volume entering the spinal canal. Proton plans significantly reduced the mean lung dose (by 21.9%), lung V5, V10, V20 (by 47.9%, 36.4%, 12.1%, respectively), mean heart dose (by 21.4%) and thoracic vertebra dose (by 29.2%) (*P* < 0.05).

**Conclusions:**

In this planning study, robust PBS plans were achievable in carefully selected patients. Considerable dose reductions to the lung, heart and thoracic vertebra were possible without compromising target coverage. Sparing these lymphopenia-related organs may be particularly important in this era of immunotherapy.

## Highlights

• Robust scanning proton plans are feasible in patients with superior sulcus tumours.
• Target coverage is comparable to volumetric-modulated arc therapy plans.
• Considerable dose reduction to the thoracic vertebra, lungs and heart is possible.
• Using an inhomogeneity correction did not improve robustness.
• The same proton and photon plans failed robustness assessment.

## Introduction

Superior sulcus tumours (SSTs) are rare subtypes of locally advanced non-small cell lung cancers (NSCLCs), representing 5% of all bronchogenic carcinomas. Outcomes are poor, with local recurrence in 40–50% of patients [[1], [2], [3]] and 5-year overall survival between 5 and 30% [4,5]. They present a unique treatment challenge by characteristically invading the chest wall and structures of the thoracic inlet, including the parietal pleura, first and second ribs and vertebral bodies; as well as (but not necessarily) the brachial plexus and stellate ganglion [[6], [7], [8]]. This makes surgical resection difficult and their close proximity to the spinal canal means that dose coverage by photon radiotherapy is often compromised. When disease is unresectable, radiotherapy (with or without chemotherapy) is the principal treatment modality.

Proton beam therapy (PBT) has a number of advantages over state-of-the-art photon-based volumetric-modulated arc therapy (VMAT). Its physical characteristics result in a relatively low entrance dose and no exit dose, potentially achieving superior target conformality while reducing dose to surrounding tissues [[9], [10], [11]]. Pencil beam scanning (PBS) is the latest technology whereby narrow proton beams are magnetically scanned across the tumour volume, promising better conformality than passively scattered protons [12]. Reservations regarding PBT, particularly PBS, are due to motion and tissue heterogeneity. These affect uncertainties in radiological path lengths [9,[13], [14], [15]] and subsequently the robustness of treatment delivery, as motion results in interplay-related dose degradation [16] and potential overdose to organs at risk (OARs).

Although an increasing number of studies investigating the use of PBT in locally advanced NSCLC have emerged over the last decade, very few have used PBS [17], the vast majority utilising passively scattered protons [[18], [19], [20], [21], [22], [23], [24], [25]]. From limited studies that do exist, PBS is suggested to better spare OARs [12,26]. A number of ongoing single-arm [[27], [28], [29]] and randomised control trials [30,31] intend to report toxicity following thoracic irradiation with PBT. The ongoing PRONTOX trial specifically aims to establish if dose-sparing translates into reduced radiation-induced toxicity [32]. Of particular interest is the potential for PBT to improve survival outcomes by sparing dose to the heart, thereby minimising the risk of cardiac toxicity and limiting dose to additional lymphopenia-related organs, such as the lungs and thoracic vertebra. Disappointingly, Liao *et al.*’s [19] recent trial reported no reduction in local failure after passively scattered protons. However, this may be due to the heterogeneity of disease stages treated (II–IV), outdated technology or inadequate image guidance at the time the study was conducted. It is clear that we have yet to identify key niche cohorts where the advantages of advanced proton techniques can be fully exploited.

Patients with SSTs seem likely candidates to benefit from scanning protons and present an opportunity to develop PBS techniques in locally advanced NSCLCs as: (i) their invasion of local structures limits motion, circumventing the challenging issues of interplay; (ii) their apical location results in smaller volumes of aerated tissue surrounding them, reducing heterogeneity along proton paths.

The aim of this retrospective planning study was to explore robust PBS planning of SSTs, assessing if target coverage is improved and if the dose to normal tissue can be significantly spared compared with VMAT.

## Materials and Methods

Patients with SSTs treated with radical radiotherapy between 2010 and 2015 were identified. All patients were positioned supine on wing boards, arms above their head and immobilised with customised vacuum bags. Four-dimensional computed tomography using the Real-time Position Management system was used to acquire a free-breathing trace during acquisition and treatment delivery.

**Fig 1:**
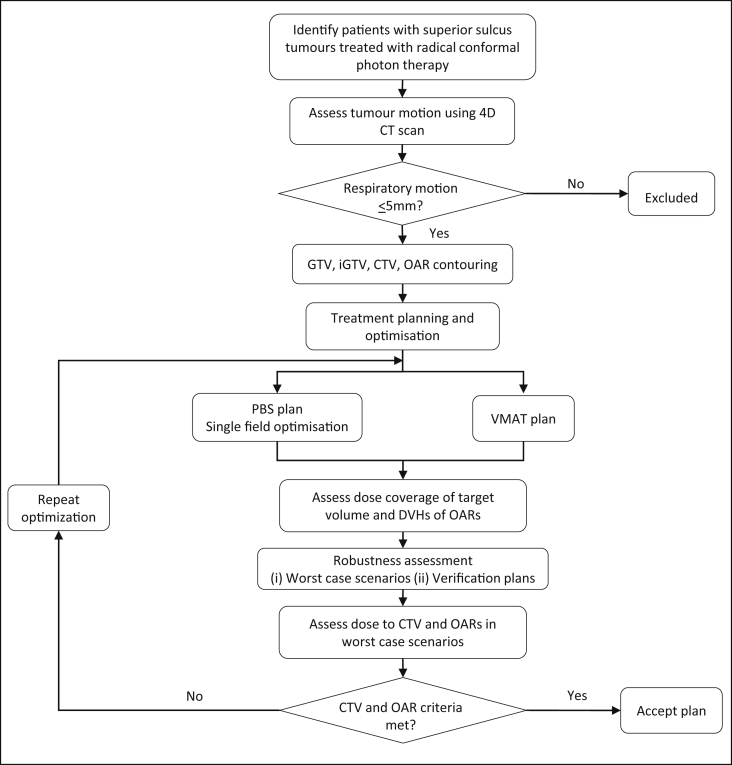
Flowchart of case selection and planning procedures. GTV, gross tumour volume; iGTV, internal gross tumour volume as assessed by four-dimensional computed tomography; CTV, clinical target volume; OAR, organs at risk; DVH, dose-volume histograms.

Patients were excluded if tumour motion was >5 mm (Figure 1). All patients were planned to 64 Gy(RBE) in 32 fractions using Eclipse (Varian Medical Systems, Palo Alto, California, USA), version: 13.7.33 for VMAT plans and version: 13.7 for proton plans.

### Motion Assessment

The range of tumour motion was verified to be ≤ 5 mm, which was considered acceptable [[33], [34], [35]] by assessing *z*-axis motion of the most inferior part of the tumour and delineating the gross tumour volume (GTV) in each phase of the four-dimensional computed tomography scan to assess centre-of-mass movement in the *x*, *y* and *z* axes.

For one test case, the OARs were also contoured on CT0 (max-inhalation) and CT50 (max-exhalation) and their centre-of-mass location noted so that their range of motion could be assessed.

### Volume Delineation

The internal GTV (iGTV) was defined as the envelope of GTV motion and delineated using the maximum-intensity projection dataset. In cases where the tumour moved into nearby soft tissues of a similar density, the maximum-intensity projection was not appropriate. Here delineation was aided by all phases, especially max-inspiration and -expiration. The clinical target volume (CTV) was defined as the iGTV + 5 mm in all directions – as such, the CTV is synonymous with the internal target volume. A technical optimisation volume was created to account for external variation in set-up, as per department tolerance guidelines, and was defined as the CTV +5 mm in all directions.

### Organ at Risk Tolerances

OARs were delineated on the average intensity projection image datasets and tolerances are defined in Supplementary Table S1 [36].

Tumour target volumes were delineated by two clinicians, one of whom delineated all the OARs. All final patient volumes were verified by a third independent clinician.

### Planning Method

VMAT plans were generated as per our departmental protocols using target and OAR structures and dose constraints, as above (see Supplementary Table S1), to set planning objectives. Two partial arcs were used to minimise unnecessary dose to normal lung.

For lung proton plans it has become commonplace to apply an inhomogeneity correction to ensure density correction and target coverage [37] but this may not be necessary for SSTs. Thus, for cases 1–3, two nominal plans were created with either an inhomogeneity correction applied to the whole iGTV (by assessing the average density within the tumour's centre and assigning a uniform CT Hounsfield unit override); or no inhomogeneity correction. Only if robustness analysis showed an advantage of applying an inhomogeneity correction would we continue for the remaining cases.

Two to three beams of equal weighting were used for all plans. Beam angles were selected with consideration of robustness and conformality. The aim was to ensure: (i) the shortest, most homogenous path to the target based on a visual check; (ii) beam entry through stable tissue; (iii) avoiding critical OARs immediately distal to the target [38]; (iv) avoiding high-Z materials (such as metal clips or prostheses). If it was felt that the more robust option resulted in compromise to conformality that was clinically unacceptable, then angles were adjusted. Beams did not overlap at the skin surface, to avoid skin toxicity, and not more than two of three of the prescribed dose came from beams directed towards a critical structure.

A single-field optimisation approach was used rather than multi-field optimisation (MFO), despite limited reports that it might have a dosimetric advantage [26] as MFO is exquisitely sensitive to motion and therefore considered potentially less robust.

The spot and layer spacing was nominally set to 5 mm.

### Optimisation Approach

Inverse plan optimisation was used for both photon and proton plans. The technical optimisation volume was used to optimise CTV coverage. The optimisation went through iterations in order to achieve OAR tolerances (highest priority) and target coverage. The target coverage assessment is described below.

Dose distribution was calculated on the average computed tomography datasets.

### Robustness Assessment

Two strategies were used to assess robustness for both VMAT and PBS plans. First, we performed robustness analysis based on worst-case scenario. A geometric uncertainty of 5 mm, based on our centre's lung set-up tolerance, was used for proton and photon plans [39]. Range uncertainty of 3.5% was considered for protons only [15,[40], [41], [42]]. The worst-case scenarios (defined as the minimum target coverage and maximum dose to OAR across the various scenarios) were assessed to ensure adequate target coverage and that dose tolerances were met. The spinal canal dose constraints were more restrictive for the nominal plan optimisation (point dose ≤46 Gy(RBE)) as a pragmatic decision to ensure it would pass robustness assessment, where up to 50 Gy(RBE) to 1 cm_3_ of the spinal canal was accepted.

Second, verification plans were calculated in order to assess the impact of motion. These were recalculations of the nominal plan on CT0 (max-inhalation) and CT50 (max-exhalation) keeping the exposure parameters constant. Dosimetric changes affecting target coverage led to further optimisation iterations and, if necessary, beam angle changes.

### Plan Evaluation

Nominal plans were considered acceptable if the CTV D95 was ≥95%, acknowledging that SST CTVs can infiltrate the spinal canal, creating a conflict of dose limitations; and OAR criteria were met.

Plans passed robustness assessment if the maximum percentage difference in target coverage (difference between CTV D95 in the worst-case scenario and CTV D95 in the nominal plan) was ≤5%; and if OAR tolerances were maintained.

### Statistical Analysis

The Mann-Whitney U test was used to calculate statistical significance (*P* values) of mean dose parameters from the VMAT compared with PBS plans using the statistics software programme R.

A value of *P* < 0.05 was considered statistically significant.

## Results

**Fig 2:**
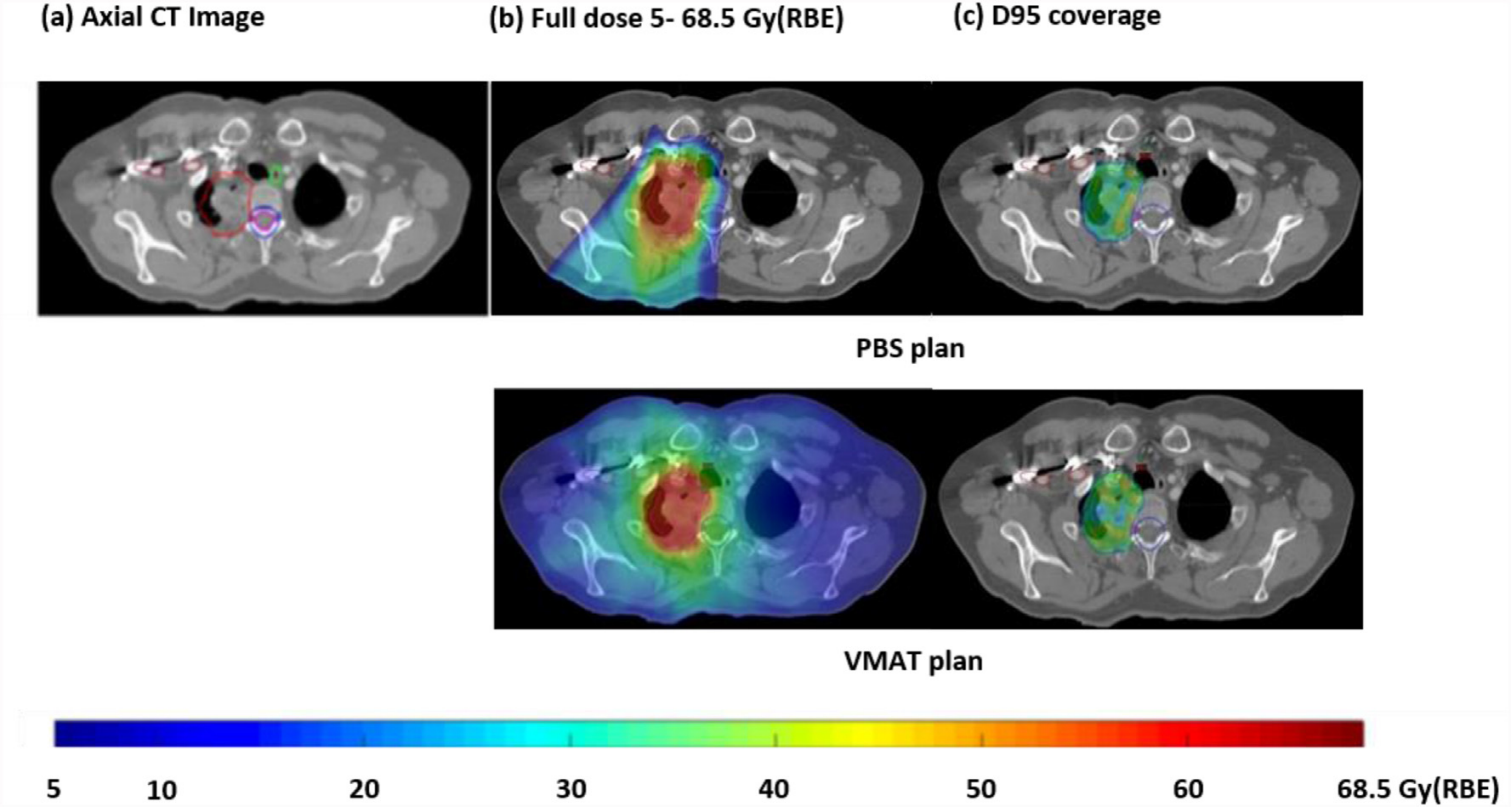
(a) Axial images, (b) full doses 5–68.5 Gy(RBE), (c) D95 coverage of case 1. The pencil beam scanning plan is shown in the top row. The volumetric-modulated arc therapy (VMAT) plan is shown in the bottom row. (a) The tumour's proximity to the spinal canal and canal planning at risk volume in each case. Structures seen include: clinical target volume (CTV, magenta), brachial plexus (red), spinal canal (pink), canal planning at risk volume (dark blue), oesophagus (light green).

Ten of 17 patients were identified as suitable – four patients were excluded as they did not have four-dimensional computed tomography scans and three had large tumours extending into the lower lobes where motion was >5 mm. The median CTV was 274.4 cm^3^ (101.4–645.8 cm^3^) (Table 1, Figure 2).

**Table 1:** Anatomical tumour characteristics for each case. All patients had histological confirmation of their diagnosis of non-small cell lung cancer. All patients had positron emission tomography-computed tomography and brain imaging as part of their staging imaging. Patient 1 had a magnetic resonance imaging thorax as additional imaging

	Patient									
	1	2	3	4	5	6	7	8	9	10
TNM stage (AJCC 7th Edition)	T3N0M0	T4N2M0	T4N0M0	T4N3M0	T3N2M0	T3N2M0	T3N2M0	T4N2M0	Locally recurrent disease	T3N3M0
Histological confirmation of diagnosis	Adenocarcinoma	Squamous cell carcinoma	Adenocarcinoma	Squamous cell carcinoma	Squamous cell carcinoma	Adenocarcinoma	Adenocarcinoma	Adenocarcinoma	Squamous cell carcinoma	Adenocarcinoma
OTV volume (cm^3^)	200.13	958.79	286.48	595.52	585.83	766.30	315.86	499.39	223.30	389.00
Tumour location	Right apex	Right apex and mediastinum	Left apex	Right apex and mediastinum	Right apex and mediastinum	Right apex and mediastinum	Right apex and mediastinum	Left apex and mediastinum	Left apex	Right apex and mediastinum
Abutting or invasion of structures	Invades mediastinal pleura medially. No brachial plexus invasion.	Involves mediastinal and peripheral pleura. Encases superior vena cava.	Abuts mediastinal and peripheral pleura. Infiltration of mediastinum at the level of the aorto pulmonary window.	Abuts second rib, four vertebral bodies and pericardial sac superiorly to bottom of pulmonary trunk	Invades third rib posteriorly	Abuts third to sixth ribs posteriorly and the T4–6 thoracic vertebral bodies anteriorly	Abuts chest wall and first to second ribs anteriorly over a longitudinal length of 2.5 cm	Invades chest wall along first to fourth ribs posteriorly	Abuts first to third ribs posteriorly and invades second rib	Abuts first to third ribs and the thoracic vertebral bodies anteriorly over a length of 2.25 cm
Minimum GTV-to- spinal canal distance (cm)	0.75	1.55	3.00	0.91	1.50	0.70	3.50	1.00	0.50	1.50
Minimum GTV-to- brachial plexus distance (cm)	Abutting the brachial plexus on one CT slice	Abutting and displacing the brachial plexus	Abutting the brachial plexus	Abutting the brachial plexus and within 0.5 cm over a length of 1.00 cm	1.54	0.40 cm at two points	Within 0.5 cm over a length of 1.50 cm	Abutting and within 0.5 cm over a length of 1.50 cm	Abutting and within 0.5 cm over a length of 1.75 cm	1.50
Minimum GTV-to- heart distance (cm)	3.25	0.25	Abutting pericardiac sac superiorly to bottom of pulmonary trunk	Abutting pericardial sac superiorly to bottom of pulmonary trunk over a length of 2.75 cm	Abutting and overlapping pericardium superiorly over a length of 5 cm	Abutting and within 0.5 cm over a length of 2.25 cm	Abutting and within 0.5 cm over a length of 5.25 cm	Abutting over a length of 3.00 cm	5.00	Abutting and within 0.5 cm over a length of 1 cm
Minimum GTV-to- oesophagus distance (cm)	2.30	1.04	Abutting for 1 cm length	Abutting and within 0.5 cm over a length of 5 cm	Within 0.5 cm from oesophagus over a length of 1.25 cm	Within 0.5 cm over a length of 1 cm	Abutting or overlapping oesophagus over a length of 4 cm	Within 0.5 cm over a length of 2 cm	Abutting and within 0.5 cm over a length of 2.75 cm	0.60
Minimum GTV-to- rib distance (cm)	Adjacent to second rib extending to the costovertebral junction but no bone invasion	Abutting first, second and third ribs posteriorly	Abutting first and second rib	Abutting second rib	Invading third rib posteriorly, tracking along second and fourth ribs posteriorly	Abutting third to sixth ribs posteriorly	Within 0.5 cm of first and second ribs anteriorly over a longitudinal length of 2.5 cm	Abutting first to fourth ribs posteriorly over a length of 5.75 cm	Abutting first to third ribs posteriorly and invading second rib	Abutting the first, second and third ribs
Minimum GTV-to- thoracic vertebrae distance (cm)	Adjacent to thoracic vertebra. Within 0.5 cm over a length of 2.50 cm	Adjacent to thoracic vertebra. Within 0.5 cm over a length of 5.25 cm	Within 1.3 cm over a length of 0.25 cm	Abutting and within 0.5 cm of the anterior part of the thoracic vertebra over a length 6.25 cm	Within 0.5 cm over a length of 0.25 cm	Abutting the anterior part of the thoracic vertebra over a length 6 cm	Within 0.5 cm over a length of 4.00 cm	Within 0.5 cm over a length of 5.00 cm	Abutting the anterior bodies of the thoracic vertebra over a length of 5.00 cm	Within 0.5 cm of the anterior bodies of the thoracic vertebrae along a length of 2.25 cm
CTV immediately adjacent to or overlapping spinal canal	Yes	No	No	No	No	Yes	No	No	Yes	No
OTV immediately adjacent to or overlapping spinal canal PRV	Yes	Yes	No	Yes	Yes	Yes	No	Yes	Yes	Yes
CTV immediately adjacent to or overlapping brachial plexus	Yes	Yes	Yes	Yes	No	Yes	Yes	Yes	Yes	No
OTV immediately adjacent to or overlapping brachial plexus	Yes	Yes	Yes	Yes	No	Yes	Yes	Yes	Yes	Yes

CT, computed tomography; CTV, clinical target volume; GTV, gross tumour volume; OTV, optimisation target volume; PRV, planning at risk volume.

### Effect of Inhomogeneity Correction on Target Coverage

Applying the inhomogeneity correction had little effect on target volume coverage and OARs. The mean CTV D95 was 98.0% (range 97.6–98.7%) compared with 98.1% (range 97.5–98.7%) with no correction. Importantly, there was minimal effect on the robustness of target coverage – a mean maximum percentage difference in CTV D95 (no inhomogeneity correction) of 1.65% (range 0.93–2.23%) compared with a mean of 3.74% (range 1.54–7.37%) when inhomogeneity correction was used (Supplementary Figure S1). Surprisingly, applying the inhomogeneity correction for case 1 made robustness worse, resulting in a CTV D95 deviation from the nominal plan of up to 7.37% (inhomogeneity correction) versus 1.79% (no inhomogeneity correction). Based on these findings, the override method was not used for the remaining seven cases.

### Dosimetric Assessment

For all plans, Dmax was <107%. CTV D95 was >97% for all cases and all the means of the dose parameters of OARs for proton plans were lower than those for VMAT plans, except heart V40 (mean: 7.1% versus 6.3%, *P* = 0.24) and brachial plexus Dmax (1 cm^3^) (mean: 62.3 Gy versus 62.0 Gy, *P* = 0.23).

**Fig 3:**
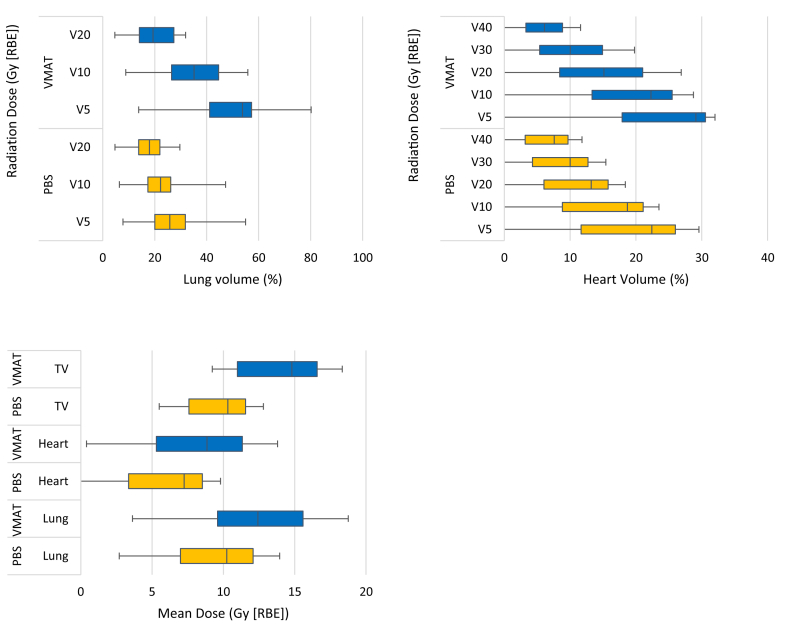
Box plot of distributions of dose–volume indices for the lungs, heart and thoracic vertebrae (TV) when planned with pencil beam scanning protons compared with volumetric-modulated arc therapy for 10 patients with superior sulcus tumours. Whiskers show range and boxes show quartiles 1, 2 and 3. Pencil beam scanning protons reduced the mean lung dose by 21.9% (mean 9.4 Gy(RBE), *P* < 0.01), lung V20 by 12.1% (mean 17.4%, *P* < 0.05), lung V10 by 36.4% (mean 21.8%, *P* < 0.01), lung V5 by 47.9% (mean 25.5%, *P* < 0.01), mean heart dose by 21.4% (mean 6.4 Gy(RBE), *P* < 0.05) and mean thoracic vertebra dose by 29.2% (mean 10.0 Gy (RBE), *P* < 0.01).

Proton plans almost completely spared the contralateral lung, on average, reducing the V5 by 79.0% (*P* < 0.01). Compared with VMAT plans, proton plans reduced the mean lung dose by 21.7% (mean 9.4 Gy(RBE), *P* < 0.01), lung V20 by 12.1% (mean 17.4%, *P* < 0.05), lung V10 by 36.4% (mean 21.8%, *P* < 0.01), lung V5 by 47.9% (mean 25.5%, *P* < 0.01), mean heart dose by 21.4% (mean 6.4 Gy(RBE), *P* < 0.05) and mean thoracic vertebra dose by 29.2% (mean 10.0 Gy(RBE), *P* < 0.01) (Table 2, Figure 3).

**Table 2:** Mean dose to target and organs at risk for the nominal photon volumetric-modulated arc therapy (VMAT) and pencil beam scanning (PBS) single-field optimisation plans. All percentage differences show a reduction in dose or volume in the PBS plans compared with the VMAT except for values preceded by (+) indicating a percentage increase in the PBS plan compared with the VMAT plan

	Assessment parameter	VMAT		PBS		Difference (%)
		Mean	Range	Mean	Range	
CTV	D95 (%)	98.4 ± 0.2	98.1–98.9	98.1 ± 0.4	97.5–98.8	0.3
	D98 (%)	97.5 ± 1.2	94.1–98.1	97.2 ± 0.6	96.2–98.2	0.3
Lung	V5 (%)	48.9 ± 15.4	13.9–68.7	25.5 ± 9.9	7.8–43.4	47.9∗∗
	V10 (%)	34.3 ± 12.8	8.9–50.2	21.8 ± 8.4	6.4–36.7	36.4∗∗
	V20 (%)	19.8 ± 8.3	4.7–29.6	17.4 ± 6.3	4.7–25.8	12.1∗
	Mean dose (Gy[RBE])	12.0 ± 4.1	3.6–16.3	9.4 ± 3.4	2.7–13.2	21.9∗∗
Contralateral lung	V5 (%)	48.6 ± 16.5	10.4–71.1	10.2 ± 15.0	0.0–49.6	79.0∗∗
	V10 (%)	27.4 ± 14.1	5.9–59.0	6.7 ± 12.2	0.0–39.9	75.4∗∗
	V20 (%)	7.4 ± 8.5	0.0–31.4	3.2 ± 7.2	0.0–23.3	56.5∗∗
Heart	V5 (%)	26.1 ± 13.2	0.0–50.3	19.3 ± 11.3	0.0–37.8	26.0
	V10 (%)	20.6 ± 10.9	0.0–41.3	15.9 ± 9.7	0.0–32.6	23.0
	V20 (%)	14.7 ± 8.1	0.0–28.0	11.8 ± 7.7	0.0–26.3	19.8
	V30 (%)	9.8 ± 5.7	0.0–17.5	9.2 ± 6.4	0.0–21.6	6.7
	V40 (%)	6.3 ± 4.0	0.0–12.3	7.1 ± 5.1	0.0–17.2	+12.8
	Mean dose (Gy[RBE])	8.1 ± 3.9	0.4–13.5	6.4 ± 4.1	0.0–14.0	21.4∗
Thoracic vertebra	Mean dose (Gy[RBE])	14.1 ± 3.4	9.2–18.1	10.0 ± 2.8	5.5–14.1	29.2∗∗
Oesophagus	V35 (%)	32.8 ± 9.9	14.6–43.1	29.8 ± 12.0	8.2–43.3	9.2
Brachial plexus	Dmax (1cm^3^)(Gy[RBE])	62.0 ± 5.0	48.2–65.0	62.3 ± 5.4	47.2–65.1	+0.5
Spinal canal	Dmax (point dose) (Gy[RBE])	43.4 ± 2.2	38.6–45.6	41.4 ± 3.6	33.6–45.3	4.6
	PRV Dmax (1cm^3^) (Gy[RBE])	45.9 ± 3.8	40.5–49.6	45.1 ± 3.9	35.6–48.3	1.6

∗*P* < 0.05. ∗∗*P* < 0.01.

CTV, clinical target volume; PRV, planning at risk volume.

### Robustness Assessment

**Fig 4:**
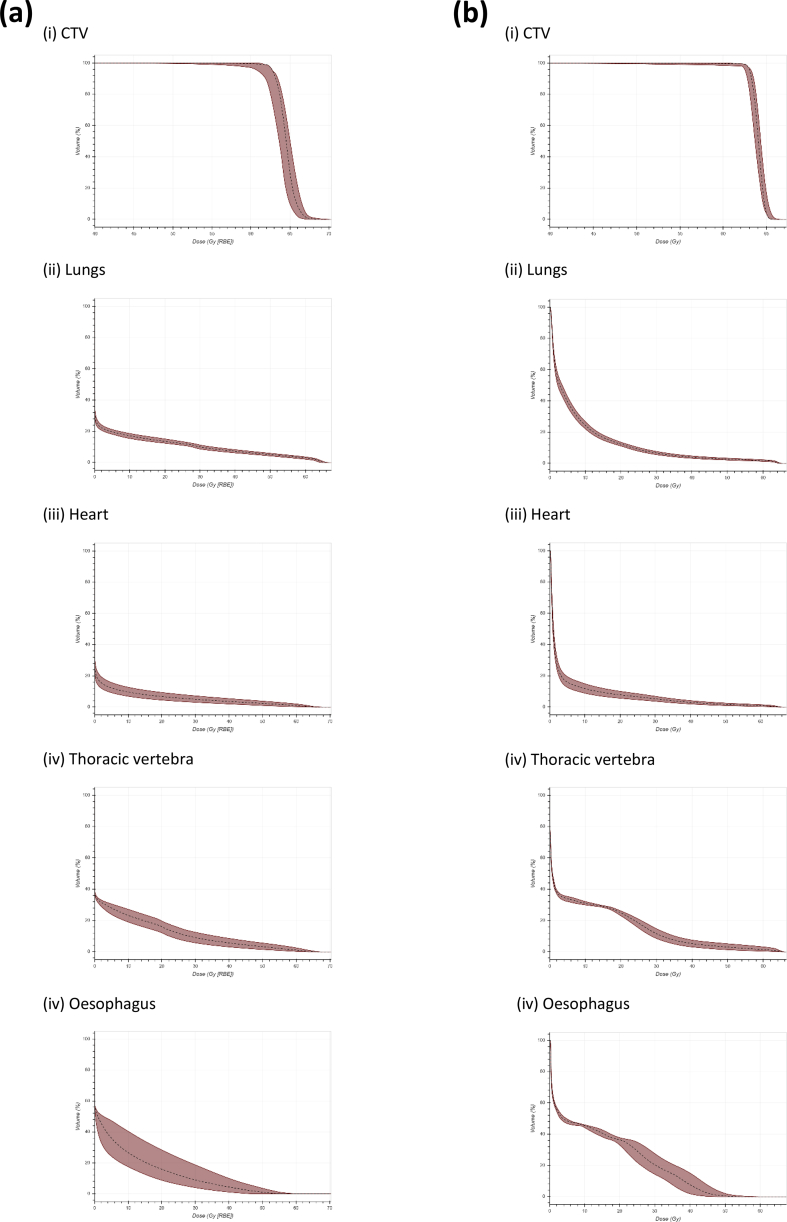
(a) Proton and (b) photon plan dose volume histograms for (i) clinical target volume (CTV), (ii) lung, (iii) heart, (iv) thoracic vertebrae and (v) oesophagus generated from robustness scenarios for patient 1 as a case example. Robustness analysis for the proton plan involved 12 scenarios of varying 5 mm isocentre shifts and ±3.5% range uncertainty. Robustness analysis for the photon plans involved six scenarios of varying 5 mm isocentre shifts.

Six scenarios were generated for the photon plans following isotropic shifts. Incorporating range uncertainty as well resulted in 12 scenarios being generated for the proton plans. Figure 4 shows the resulting CTV and OAR variation for case 1 as an example. On robustness analysis, the mean CTV D95, in the worst-case scenario, was 93.9% ± 3.0 (range 89.5–97.8%) for proton plans (maximum percentage difference 0.9–9.0%) and 97% ± 1.3 (range 93–98%) for VMAT plans (maximum percentage difference 0.26–5.49%). For all photon and proton verification plans, the CTV D95 was >95%, except the proton plan for case 9 where CTV D95 was 91.9%.

Relative OAR motion at the extremes of breathing on the test case was <3 mm, except for the heart and lungs (see Supplementary Table S2). As such, OAR dosimetry on verification plans were not carried out, especially as uncertainty scenarios identified breaches in OAR constraints.

The same four proton and photon plans failed robustness assessment (Table 3). Cases planned using VMAT primarily failed due to lung V20 tolerance being exceeded in one scenario, whereas inadequately robust CTV coverage was the dominant reason for the proton-planned cases not passing assessment. Both VMAT and PBS plans failed robustness assessment for case 9, probably a result of the CTV abutting the spinal cord.

**Table 3:** Volumetric-modulated arc therapy (VMAT) and pencil beam scanning (PBS) plans that failed robustness assessment

Case	VMAT	PBS
	Reason(s) for failing robustness assessment	Reason(s) for failing robustness assessment
2	Lung V20 tolerance exceeded on one worst-case scenario from robustness analysis	Spinal canal tolerance exceeded on one worst-case scenario from robustness analysis
4	Lung V20 tolerance exceeded on one worst-case scenario from robustness analysis	Maximum percentage difference in CTV coverage on robustness analysis >5%
7	Lung V20 tolerance exceeded on one worst-case scenario from robustness analysis	Maximum percentage difference in CTV coverage on robustness analysis >5%
9	Maximum percentage difference in CTV coverage on robustness analysis >5%	Maximum percentage difference in CTV coverage on robustness analysis >5%

CTV, clinical target volume.

## Discussion

Our results suggest that it is feasible to deliver robust PBS treatment in select SST cases where tumour motion ≤5 mm and tissue heterogeneity along the proton paths is minimal. Comparable CTV coverage and considerable reduction in lung, mean heart dose and mean thoracic vertebra dose can be achieved.

SSTs present similar challenges for photons and protons. These include the image quality needed for accurate tumour and OAR delineation, motion and the proximity of critical organs. Additional considerations for protons are inherent uncertainties, such as range, lateral scattering and exquisite sensitivity to changes in anatomy [9,[13], [14], [15]].

Magnetic resonance imaging (MRI) helps to evaluate SSTs, as higher contrast resolution results in superior anatomical visualisation, especially the brachial plexus. MRI-guided radiotherapy (MR-linac) is an evolving technology incorporating MRI sequences to improve delineation accuracy. It can enable uncertainty margins to be reduced, thus better sparing OARs [43]. However, respiratory motion can cause ghosting artefacts and compromise resolution [44]. Furthermore, although advanced MRI-to-four-dimensional computed tomography registration algorithms exist, they are not yet used in clinical practice [45] and assessment on an individual basis, in order to minimise error propagation, is recommended [46]. Studies comparing MR-linac and PBT for SSTs should be explored.

Motion is problematic for any form of high-precision radiotherapy where steep dose gradients can result in dose uncertainty. Our motion monitoring strategy relied on external devices tracking chest wall movement but this can be smaller than that of the tumour [13,47]. Subsequent analysis assessed overall and maximal motion but rotational or tumour deformity analysis was not possible. Our study only included apical tumours with motion ≤5 mm, therefore negating the need to evaluate interplay [[33], [34], [35],^48^]. The impact of motion on dosimetry was analysed by robustness assessment and considered for both VMAT and PBS. To our knowledge, this has not been carried out in previous comparative lung planning studies.

Protons are particularly sensitive to motion as their radiological path length is affected, not only by tumour movement but also normal lung tissue – at different phases of breathing, variable filling of airways and blood vessels results in variable relative stopping power ratio values [40]. This interplay between the scanned beam and target motion not only causes degradation of dose homogeneity, due to misplacement of individual spots relative to planned positions, but it also affects the dose to critical organs [15,49,50]. Dose-repainting has been proposed to reduce this, but it is not effective alone [33,50] and questions remain about the effect of washout [16,33,48,51].

Our proton planning approach focused on maximising robustness. Considerations included defining margins, choosing robust beam angles and investigating the use of an inhomogeneity correction. The concept of beam-specific margins that incorporate proximal and distal uncertainties has been implemented in passive scattering protons but the translation to PBS is not well established [52] so was not applied here. Another strategy to allow for uncertainties is to use minimax optimisation [53]. Here, uncertainties are entered into the planning system and multiple scenario-based plans are generated. This bypasses the need for a technical optimisation volume and coverage is thought to be equivalent to technical optimisation volume-based plans [[54], [55], [56]]. Although it is recognised that technical optimisation volume margins effectively only take lateral uncertainties into account, we compensated for this by retrospectively assessing target coverage before re-optimising areas of under-coverage [57].

All proton plans were achieved using a two-to three-field arrangement, which is in keeping with previous studies [10,26,[58], [59], [60]] who have used between two and four beams. In principle, an increased number of beams increases robustness, at the expense of an increased integral dose and dose to certain OARs.

The main purpose of an inhomogeneity correction is to account for motion-induced tissue density variation to minimise the risk of under-dosing the target and is widely used in passively scattered techniques [50,61,62]. However, this is an artificial scenario and our study showed that its effect on plan robustness was minimal, probably due to SSTs being more fixed and their apical location. Unexpectedly, the inhomogeneity correction resulted in reduced CTV robustness for one case due to the complex geometry and relative position of the tumour limiting choices of beam angles. This meant part of a beam was directed through the shoulder blade. Under uncertainties, the amount of bone in the path increased. The effect was less detrimental when no inhomogeneity correction was used as the extra bone was counteracted by more lung in the path length. This extra lung was overridden with the inhomogeneity correction and resulted in under-coverage being observed. On visual assessment, it was clear that the inhomogeneity correction effect became more noticeable in parts of tumours that were spiculated and extended lower into the thorax where movement was greater. The most important factor affecting target coverage and robustness was the target volume overlapping with the spinal canal. The need to compromise target coverage in order to adhere to spinal canal tolerance remains as much a problem for PBS protons as it does for VMAT. This lack of improved target conformality may be explained by the lateral penumbra for PBS being worse than for photons and range straggling blunting the sharpness of distal dose fall-off such that safety margins are still needed [63]. In contrast to other reports [21], our results did not show size affecting target coverage or robustness.

Robustness analysis tools within treatment planning systems are commonly used for the assessment of proton plans [12] and can be applied to photon plans [39]. In Chang *et al.*’s [12] study comparing PBS-MFO, passive scattering protons and intensity-modulated radiotherapy (IMRT) for thoracic tumours, robustness was assessed by verification plans and nine scenarios of ±3 mm shifts (although set-up error was 5 mm) and ±3.5% range uncertainty, with a prescription criteria of CTV D95 > 95%. They concluded that MFO enabled a dosimetric advantage in sparing OARs but no advantage in CTV coverage [12]. Similarly, we used a combination 12 scenarios with various permutations of ±5 mm isocentre shifts and ±3.5% range uncertainty [40] as well as verification plans at CT0 and CT50. In this way, target coverage and OAR doses could be assessed in worst-case scenarios and at the extremes of the breathing cycle – a necessary two-step check. Verification plans on repeat computed tomography datasets were not calculated, however, so the impact of inter-fractional rotational variation was not accessed with this initial cohort.

The same four cases failed robustness assessment for photon and proton planning – the photon plans primarily due to lung V20 tolerance being exceeded and the proton plans due to CTV D95 variation being unacceptable, highlighting the specific challenges faced by both techniques. It was not possible to create VMAT or PBS plans that robustly covered the CTV for case 9, emphasising that the most critical factor affecting target robustness was the CTV entering the spinal canal. One proton-planned case failed due to an uncertainty scenario where the isocentre shift resulted in the spinal canal being placed into the beam's path resulting in an excess dose to it. In reality, such a scenario would be avoided by ensuring patients are shifted to ‘0’ position following verification imaging prior to each fraction – this technique is applied to all patients with tumours close to the spinal canal. These results show that it is not practically possible to resolve all uncertainties in the planning phase and it does not diminish the need for diligent image guidance and adaptive strategies during treatment.

The most significant dosimetric advantage of protons was in sparing central structures such as the heart and thoracic vertebra (in addition to lungs), suggesting that SSTs with associated mediastinal involvement will probably show the greatest benefit from PBS. There is accumulating evidence correlating low dose to lungs and heart to poorer survival [64]. This is thought to be a result of irradiation of circulating lymphocytes [[64], [65], [66], [67]], as well as cardiac toxicity. Tang *et al.* [66] showed significant correlation between lung V5–10 and lymphocyte nadir and Joseph *et al.* [68] showed in their retrospective analysis that higher integral heart doses correlated with a decline in post-treatment lymphocyte counts. Additionally, limiting dose to the thoracic vertebra, where 35% of haematopoietic bone marrow is located, also reduces the risk of lymphopenia. Based on previous studies, if the lung V5, V10, V20 and mean thoracic vertebra dose are kept under about 65%, 55%, 45% and 23 Gy, respectively, the risk of grade 3 (or higher) haematological toxicity can be dramatically reduced [[69], [70], [71]]. We showed a significant reduction in dose to these lymphopenia-related organs, which may be the most advantageous role of PBS-PBT in this era of immunotherapy.

### Limitations

The Monte Carlo calculation algorithm is considered to be more accurate in heterogeneous environments, like the lung cohort [72]. As expected, Monte Carlo calculations had little effect on the OARs, but showed an approximate 5% reduction in mean CTV D95 compared with the clinical algorithm utilised, which is in agreement with other reports [73,74]. Unfortunately, Monte Carlo-based optimisation is not currently available within the Eclipse treatment planning system but follow-up studies utilising this are warranted.

## Conclusions

In our planning study, we demonstrated that robust PBS plans are achievable in carefully selected patients. Significant dose reductions to the lung, heart and thoracic vertebra are possible without compromising target coverage. Sparing these lymphopenia-related organs may be particularly important in this era of immunotherapy. Identifying suitable cases that would probably benefit from scanning protons is crucial and further analyses on a larger patient cohort is required.

## Conflicts of Interest

Dr R.A. Sharma previously held consultancies and research grants as listed (within the last 3 years). He reports personal fees from Affidea, personal fees from 10.13039/100004325AstraZeneca, personal fees from 10.13039/100008497Boston Scientific, personal fees from 10.13039/100011968Cetacean Research Technology, personal fees from 10.13039/501100003769Eisai, personal fees from 10.13039/501100008645Terumo, grants, personal fees and other from Sirtex Medical, grants, personal fees and other from 10.13039/100014869BTG, outside the submitted work. He is currently an employee of Varian Medical Systems. R. Amos reports grants from 10.13039/100007210Varian, personal fees from TAE Life Sciences, personal fees from 10.13039/100007566City, University of London and personal fees from 10.13039/501100001261London South Bank University, outside the submitted work. Dr S. Wong, J. Alshaikhi, H. Grimes, A. Poynter, V. Rompokos, Dr S. Gulliford, Dr G. Royle, Dr Z. Liao, and Dr R. Mendes declare no competing interests.
