# Supplementary material for: Retrospective Planning Study of Patients with Superior Sulcus Tumours Comparing Pencil Beam Scanning Protons to Volumetric-Modulated Arc Therapy
Source: Clin Oncol (R Coll Radiol). 2021 Mar;33(3):e118–31. doi: 10.1016/j.clon.2020.07.016 (PMC7883303; doi:10.1016/j.clon.2020.07.016)
Supplement: Multimedia component 1 [file mmc1.pptx]

## Slide 1
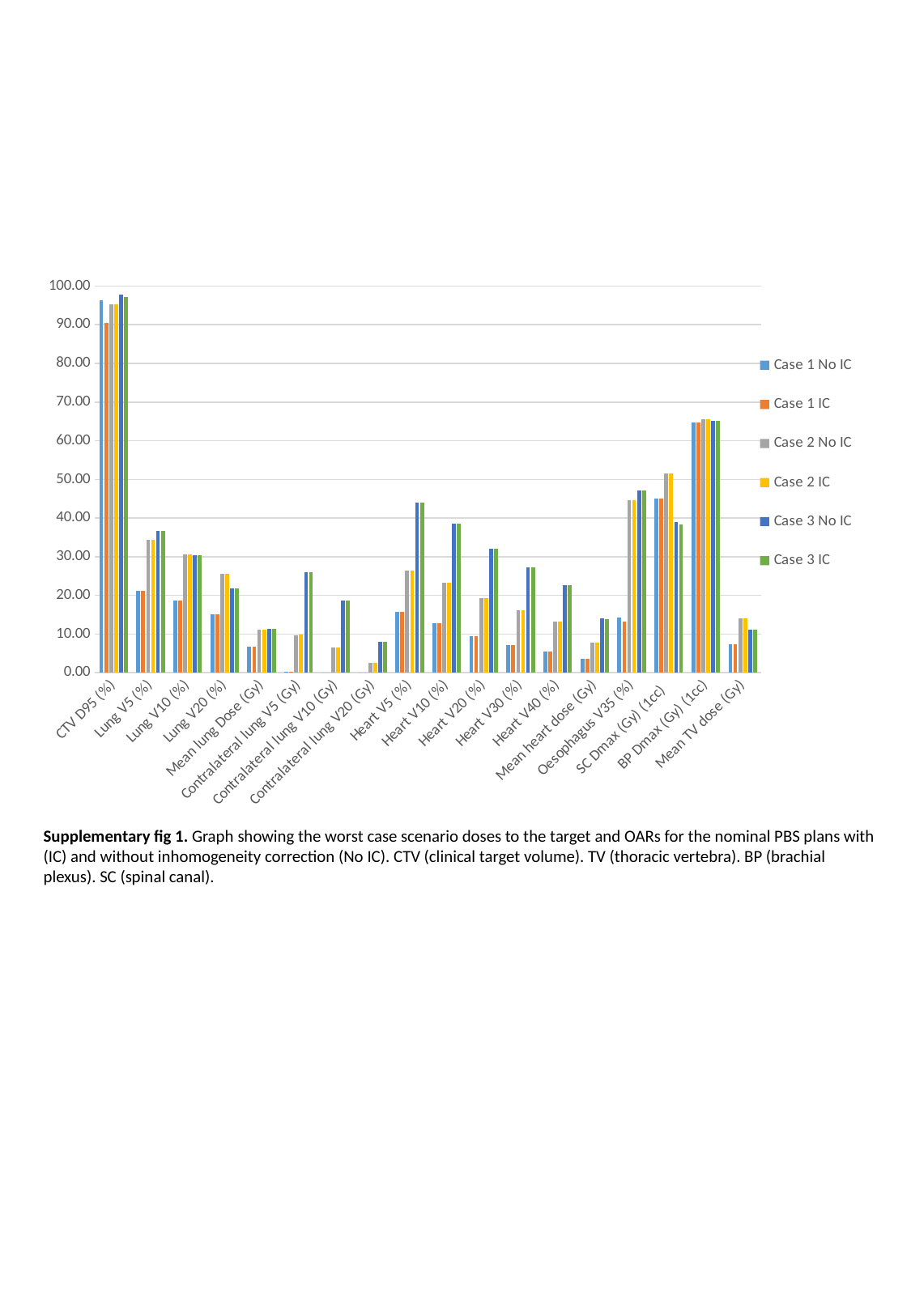

### Chart
| Category | Case 1 | Case 1 | Case 2 | Case 2 | Case 3 | Case 3 |
|---|---|---|---|---|---|---|
| CTV D95 (%) | 96.34 | 90.47 | 95.33 | 95.32 | 97.78 | 97.13 |
| Lung V5 (%) | 21.14 | 21.14 | 34.28 | 34.29 | 36.65 | 36.64 |
| Lung V10 (%) | 18.59 | 18.59 | 30.67 | 30.67 | 30.46 | 30.46 |
| Lung V20 (%) | 15.12 | 15.12 | 25.55 | 25.55 | 21.71 | 21.7 |
| Mean lung Dose (Gy) | 6.74 | 6.73 | 11.14 | 11.14 | 11.4 | 11.37 |
| Contralateral lung V5 (Gy) | 0.2 | 0.19 | 9.7 | 9.86 | 25.97 | 25.97 |
| Contralateral lung V10 (Gy) | 0.0 | 0.0 | 6.45 | 6.45 | 18.58 | 18.56 |
| Contralateral lung V20 (Gy) | 0.0 | 0.0 | 2.51 | 2.51 | 8.07 | 8.06 |
| Heart V5 (%) | 15.77 | 15.75 | 26.47 | 26.46 | 44.07 | 44.05 |
| Heart V10 (%) | 12.72 | 12.72 | 23.31 | 23.31 | 38.6 | 38.6 |
| Heart V20 (%) | 9.39 | 9.39 | 19.27 | 19.27 | 32.05 | 32.05 |
| Heart V30 (%) | 7.22 | 7.22 | 16.07 | 16.06 | 27.18 | 27.15 |
| Heart V40 (%) | 5.46 | 5.46 | 13.26 | 13.26 | 22.67 | 22.61 |
| Mean heart dose (Gy) | 3.66 | 3.66 | 7.75 | 7.75 | 13.95 | 13.9 |
| Oesophagus V35 (%) | 14.2 | 13.31 | 44.59 | 44.59 | 47.22 | 47.21 |
| SC Dmax (Gy) (1cc) | 45.05 | 45.0 | 51.59 | 51.57 | 39.02 | 38.3 |
| BP Dmax (Gy) (1cc) | 64.79 | 64.79 | 65.57 | 65.57 | 65.18 | 65.23 |
| Mean TV dose (Gy) | 7.44 | 7.44 | 14.13 | 14.14 | 11.16 | 11.03 |Supplementary fig 1. Graph showing the worst case scenario doses to the target and OARs for the nominal PBS plans with (IC) and without inhomogeneity correction (No IC). CTV (clinical target volume). TV (thoracic vertebra). BP (brachial plexus). SC (spinal canal).
