# Supplementary material for: Retrospective Planning Study of Patients with Superior Sulcus Tumours Comparing Pencil Beam Scanning Protons to Volumetric-Modulated Arc Therapy
Source: Clin Oncol (R Coll Radiol). 2021 Mar;33(3):e118–31. doi: 10.1016/j.clon.2020.07.016 (PMC7883303; doi:10.1016/j.clon.2020.07.016)
Supplement: Multimedia component 2 [file mmc2.pptx]

## Slide 1
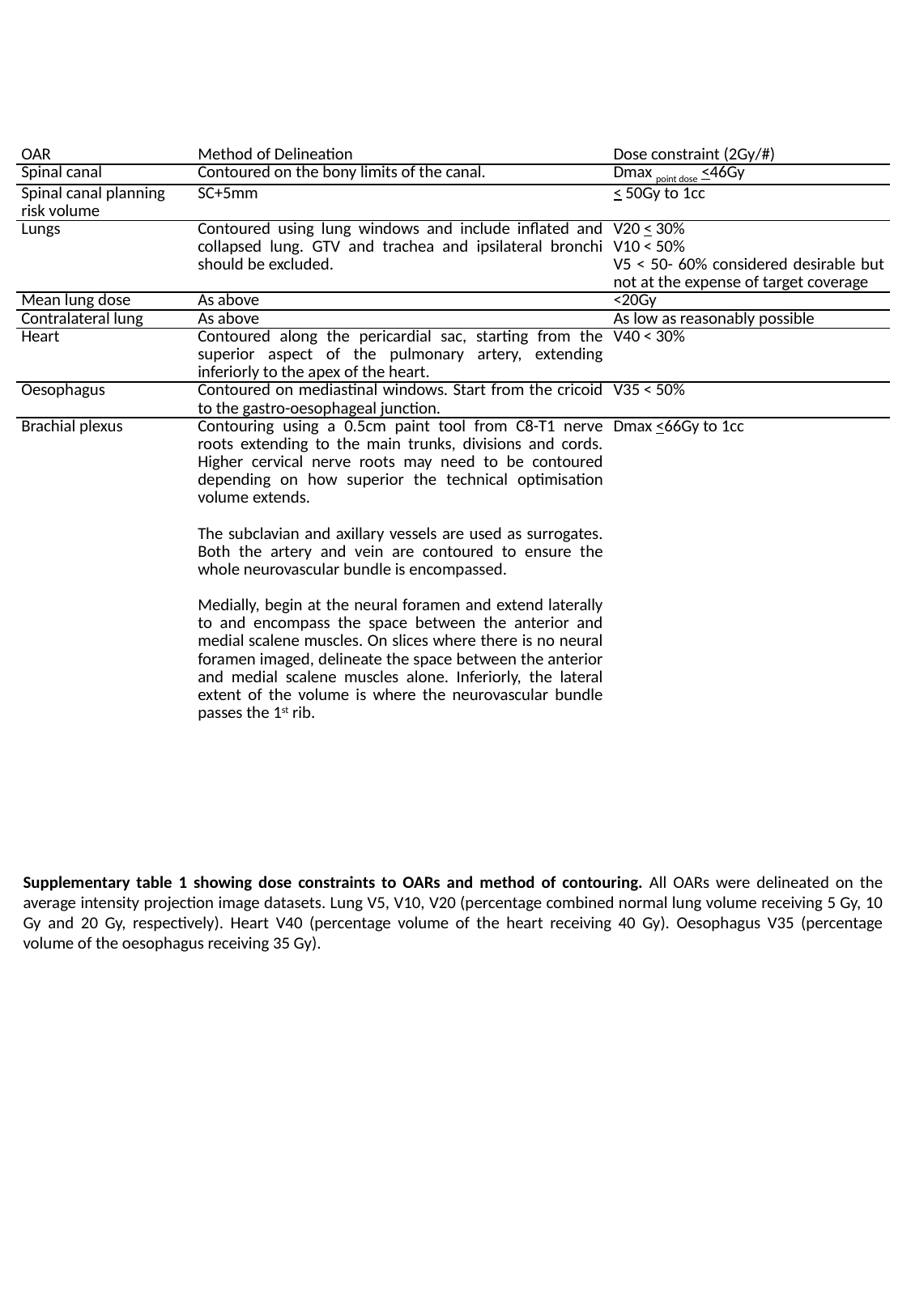

| OAR | Method of Delineation | Dose constraint (2Gy/#) |
| --- | --- | --- |
| Spinal canal | Contoured on the bony limits of the canal. | Dmax point dose <46Gy |
| Spinal canal planning risk volume | SC+5mm | < 50Gy to 1cc |
| Lungs | Contoured using lung windows and include inflated and collapsed lung. GTV and trachea and ipsilateral bronchi should be excluded. | V20 < 30% V10 < 50% V5 < 50- 60% considered desirable but not at the expense of target coverage |
| Mean lung dose | As above | <20Gy |
| Contralateral lung | As above | As low as reasonably possible |
| Heart | Contoured along the pericardial sac, starting from the superior aspect of the pulmonary artery, extending inferiorly to the apex of the heart. | V40 < 30% |
| Oesophagus | Contoured on mediastinal windows. Start from the cricoid to the gastro-oesophageal junction. | V35 < 50% |
| Brachial plexus | Contouring using a 0.5cm paint tool from C8-T1 nerve roots extending to the main trunks, divisions and cords. Higher cervical nerve roots may need to be contoured depending on how superior the technical optimisation volume extends.   The subclavian and axillary vessels are used as surrogates. Both the artery and vein are contoured to ensure the whole neurovascular bundle is encompassed.   Medially, begin at the neural foramen and extend laterally to and encompass the space between the anterior and medial scalene muscles. On slices where there is no neural foramen imaged, delineate the space between the anterior and medial scalene muscles alone. Inferiorly, the lateral extent of the volume is where the neurovascular bundle passes the 1st rib. | Dmax <66Gy to 1cc |
Supplementary table 1 showing dose constraints to OARs and method of contouring. All OARs were delineated on the average intensity projection image datasets. Lung V5, V10, V20 (percentage combined normal lung volume receiving 5 Gy, 10 Gy and 20 Gy, respectively). Heart V40 (percentage volume of the heart receiving 40 Gy). Oesophagus V35 (percentage volume of the oesophagus receiving 35 Gy).
