# Supplementary material for: Retrospective Planning Study of Patients with Superior Sulcus Tumours Comparing Pencil Beam Scanning Protons to Volumetric-Modulated Arc Therapy
Source: Clin Oncol (R Coll Radiol). 2021 Mar;33(3):e118–31. doi: 10.1016/j.clon.2020.07.016 (PMC7883303; doi:10.1016/j.clon.2020.07.016)
Supplement: Multimedia component 3 [file mmc3.pptx]

## Slide 1
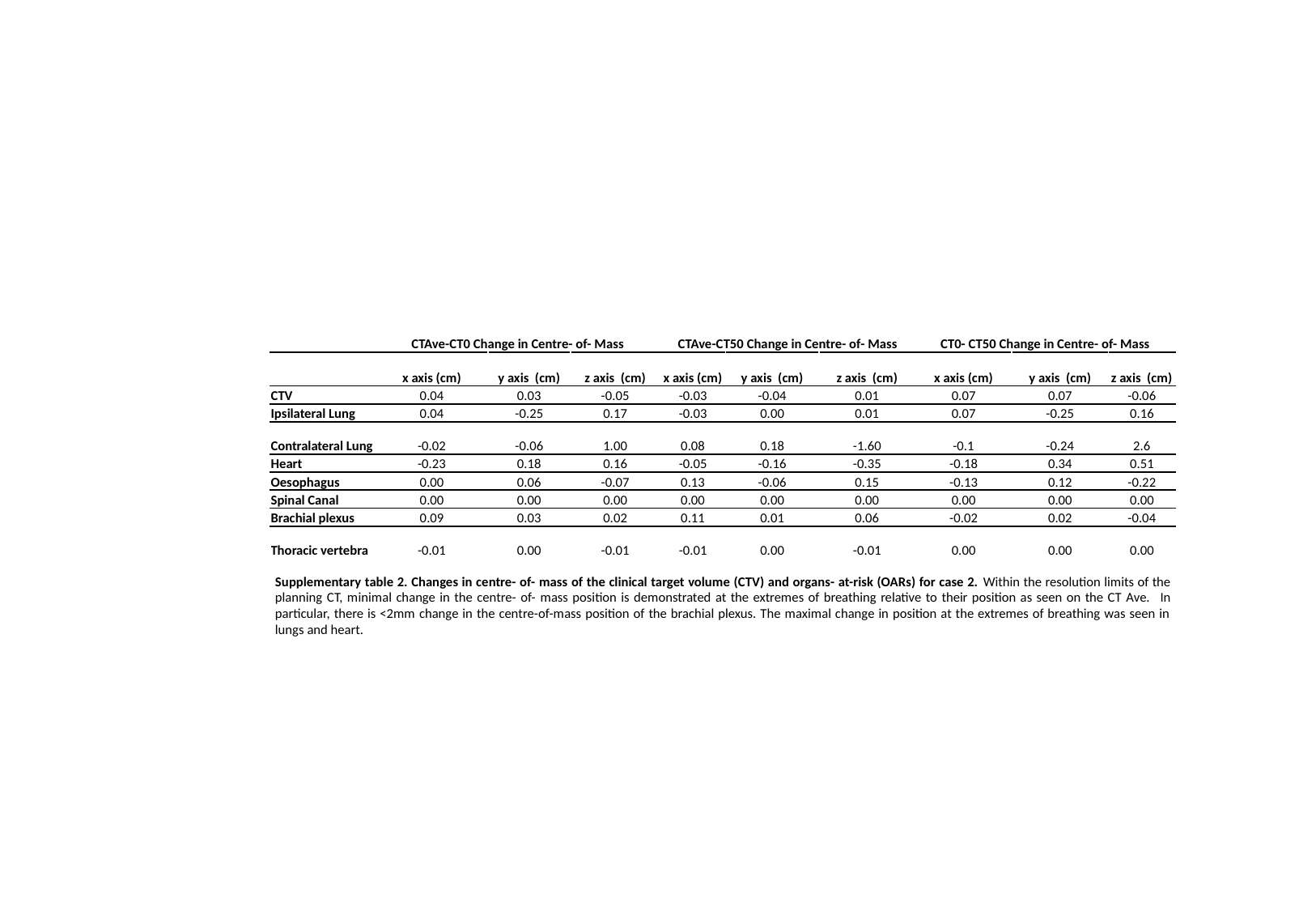

| | CTAve-CT0 Change in Centre- of- Mass | | | CTAve-CT50 Change in Centre- of- Mass | | | CT0- CT50 Change in Centre- of- Mass | | |
| --- | --- | --- | --- | --- | --- | --- | --- | --- | --- |
| | x axis (cm) | y axis (cm) | z axis (cm) | x axis (cm) | y axis (cm) | z axis (cm) | x axis (cm) | y axis (cm) | z axis (cm) |
| CTV | 0.04 | 0.03 | -0.05 | -0.03 | -0.04 | 0.01 | 0.07 | 0.07 | -0.06 |
| Ipsilateral Lung | 0.04 | -0.25 | 0.17 | -0.03 | 0.00 | 0.01 | 0.07 | -0.25 | 0.16 |
| Contralateral Lung | -0.02 | -0.06 | 1.00 | 0.08 | 0.18 | -1.60 | -0.1 | -0.24 | 2.6 |
| Heart | -0.23 | 0.18 | 0.16 | -0.05 | -0.16 | -0.35 | -0.18 | 0.34 | 0.51 |
| Oesophagus | 0.00 | 0.06 | -0.07 | 0.13 | -0.06 | 0.15 | -0.13 | 0.12 | -0.22 |
| Spinal Canal | 0.00 | 0.00 | 0.00 | 0.00 | 0.00 | 0.00 | 0.00 | 0.00 | 0.00 |
| Brachial plexus | 0.09 | 0.03 | 0.02 | 0.11 | 0.01 | 0.06 | -0.02 | 0.02 | -0.04 |
| Thoracic vertebra | -0.01 | 0.00 | -0.01 | -0.01 | 0.00 | -0.01 | 0.00 | 0.00 | 0.00 |
Supplementary table 2. Changes in centre- of- mass of the clinical target volume (CTV) and organs- at-risk (OARs) for case 2. Within the resolution limits of the planning CT, minimal change in the centre- of- mass position is demonstrated at the extremes of breathing relative to their position as seen on the CT Ave. In particular, there is <2mm change in the centre-of-mass position of the brachial plexus. The maximal change in position at the extremes of breathing was seen in lungs and heart.
